# Editorial of Special Issue “Molecular Research and Recent Advances in Diabetic Retinopathy: Second Edition”

**DOI:** 10.3390/biomedicines14050979

**Published:** 2026-04-24

**Authors:** Tomislav Bulum, Martina Tomić

**Affiliations:** 1 Department of Diabetes and Endocrinology, Vuk Vrhovac University Clinic for Diabetes, Endocrinology and Metabolic Diseases, Merkur University Hospital, Dugi dol 4a, 10000 Zagreb, Croatia; 2 School of Medicine, University of Zagreb, Šalata 3, 10000 Zagreb, Croatia; 3 Department of Ophthalmology, Vuk Vrhovac University Clinic for Diabetes, Endocrinology and Metabolic Diseases, Merkur University Hospital, Dugi dol 4a, 10000 Zagreb, Croatia; martina.tomic@kb-merkur.hr

Diabetes is a global pandemic and, with its chronic complications, represents a major health issue worldwide, as 12.2% of all global deaths in 2021 were related to diabetes [1]. In 2024, there were 589 million adults living with diabetes, with projections indicating that this number will rise to 853 million by 2050 [1,2]. Diabetes was responsible for 3.4 million deaths in 2024 and accounted for at least USD 1 trillion in health expenditure, which is a 338% increase over the last 17 years [2].

Diabetic retinopathy (DR), a microvascular complication of diabetes, is a leading cause of preventable blindness, particularly among working-age adults, due to the harmful effects of hyperglycemia on small vessels and neuroretinal structures [3]. It is estimated that approximately 75% of patients with type 1 diabetes and 25% of those with type 2 diabetes will develop DR, with about 10% experiencing vision-threatening forms such as proliferative DR (PDR) and diabetic macular edema (DME) [4]. To mitigate the impact of DR and its vision-threatening stages, early diagnosis in the asymptomatic phase is crucial to allow timely initiation of appropriate therapy. Consequently, several countries have introduced screening programs, including AI-based automated systems for DR detection [5]. Although there has been a recent decline in diabetes-related blindness with the introduction of novel treatments such as anti-vascular endothelial growth factor (anti-VEGF) agents, the risk of DR complications remains unacceptably high [6,7]. In addition to optimal glucose control, effective management of other metabolic risk factors and regular screening remain key strategies for preventing the development and progression of DR [8,9].

Molecular research in DR has substantially improved our understanding of the complex mechanisms underlying this vision-threatening complication of diabetes [10]. Chronic hyperglycemia induces oxidative stress, inflammation, and microvascular dysfunction, which contribute to retinal neurodegeneration and breakdown of the blood–retinal barrier. Key molecular pathways implicated in DR include VEGF signaling, advanced glycation end-product (AGE) formation, protein kinase C activation, and dysregulation of inflammatory cytokines [11,12]. Recent studies have also highlighted the role of epigenetic modifications, non-coding RNAs, and metabolic memory in the progression of retinal damage [13]. Advances in transcriptomics, proteomics, and metabolomics have enabled the identification of novel biomarkers and therapeutic targets. Furthermore, molecular insights have supported the development of targeted therapies, including anti-VEGF agents and emerging treatments aimed at inflammation and neuroprotection. Continued molecular research is essential for improving early detection strategies and developing more personalized and disease-modifying interventions for DR.

With all this in mind, additional efforts are needed to reduce the risk of blindness and disability in patients with diabetes. This Special Issue aims to present the latest molecular research and recent advances in DR and DME, comprising five research articles, one review, one systematic review, and one editorial.

The first research article investigated the association between the thickness of the macular ganglion cell–inner plexiform layer (GC-IPL) and DR in patients with type 2 diabetes, as well as the related risk factors. The GC-IPL is a recognized marker of retinal neurodegeneration, and alterations in GC-IPL thickness have been implicated in the development and progression of DR, with the rate of GC-IPL thinning reported to be significantly faster in patients with diabetes [14,15]. The results of this study, which included 50 eyes, demonstrated a negative correlation between macular GC-IPL thickness and DR severity in type 2 diabetes, associated with diabetes duration and glycated hemoglobin levels. The optimal predictive model for GC-IPL thickness included serum lipid levels and the presence of DR [16]. These findings support the concept that DR involves not only vascular damage but also neurodegenerative processes, emphasizing that neurodegeneration is an important component of the disease that has traditionally been regarded primarily as vascular in nature.

When it comes to the second research article, it proposed a novel ensemble meta-model for enhanced retinal blood vessel segmentation using deep learning architectures. Retinal blood vessels provide critical information for detecting various ocular diseases such as DR, glaucoma, retinal hemorrhage, and hypertensive retinopathy, while images of the macular region offer important details for identifying these conditions [17]. However, due to the complex morphology of the vascular structure and other imaging limitations, accurate segmentation can be challenging. To address this problem, the study proposed a novel ensemble learning framework that combines four deep learning architectures: ResNet50, U-Net, U-Net with a ResNet50 backbone, and U-Net with a transformer block. The ensemble meta-model demonstrated state-of-the-art performance and high accuracy in segmenting thin and complex vessel structures, particularly under conditions of noise and poor visibility [18].

The third research article investigated microvascular lesions in a large real-world dataset to validate their use as a precise tool for classifying the severity of DR. The researchers analyzed more than 2000 retinal fundus images of patients with diabetes obtained from four international datasets (Spain, India, China, and the United States). Advanced automated image analysis techniques were applied to central retinal images to quantify specific microvascular lesions—microaneurysms, hemorrhages, and hard exudates—and to validate reliable metrics for assessing DR severity. It is well known that chronic hyperglycemia in diabetes triggers a cascade of microvascular changes that begins with microaneurysm formation and progresses to hemorrhages and hard exudates [19]. The researchers in this study observed a statistically significant increase in the number of microvascular lesions and emphasized the importance and potential of lesion counting as a key biomarker for classifying and assessing DR severity [20].

As for the fourth research article, it explores the metabolic, microvascular, and structural predictors of long-term functional changes evaluated by multifocal electroretinography in patients with type 1 diabetes. Multifocal electroretinography detects retinal bioelectrical responses and can identify subtle functional alterations even before clinical manifestations of DR appear [21]. This prospective observational cohort study included 22 patients with type 1 diabetes and mild nonproliferative DR who were followed for four years using multimodal imaging approaches, including color fundus photography, optical coherence tomography (OCT), OCT angiography, and adaptive optics. By combining standard imaging techniques such as OCT, OCT angiography, adaptive optics, and multifocal electroretinography, the researchers confirmed that alterations in microvascular perfusion and photoreceptor organization are closely related to retinal function. The involvement of the choriocapillaris appears to be one of the most important underlying factors in DR progression, influencing the structural reorganization of the retina from the earliest stages of the disease. Moreover, hyperglycemia was identified as the key risk factor affecting photoreceptor function [22].

The final research article investigated retinochoroidal vascular changes assessed by OCT angiography in patients with long duration of disease but without DR. OCT angiography has shown considerable potential in detecting early retinal structural and functional changes associated with DR [23]. This study compared seventy-eight eyes from well-controlled patients with type 1 diabetes and over 15 years of disease duration with eyes from 130 healthy subjects. Researchers found microvascular abnormalities affecting retinal and choriocapillaris blood perfusion, along with anatomical changes in retinal blood vessels in long-standing, well-controlled patients with type 1 diabetes without DR. Those abnormalities included a decrease in blood flow in all studied areas of the superficial capillary plexus and in all locations except the central one of the deep capillary plexus. Choriocapillaris blood perfusion was found to be increased in the central area in patients with type 1 diabetes compared to healthy controls, while the flow in the superficial capillary plexus was correlated with well-known risk factors: microalbuminuria and duration of diabetes [24].

The review article provides an in-depth investigation of liquid biopsy combined with multi-omics approaches in the diagnosis, management, and progression of DR. Liquid biopsy is a minimally invasive diagnostic tool that allows analysis of different biomaterials like vitreous humor, aqueous humor, tears, and blood as an alternative method in the diagnosis, prognosis, management, and progression of DR [25]. This review highlights the advances in liquid biopsy techniques and sample collection methods and investigates important protein biomarkers such as inflammatory biomarkers, vascular dysfunction biomarkers, neurodegeneration biomarkers, and metabolic dysregulation biomarkers. Authors also explored the associations between different biofluids to identify biomarkers connected to the pathogenesis of DR and tried to personalize the clinical approach. Nowadays, artificial intelligence might help in analyzing multi-omics liquid biopsy data and complex biomarker patterns to conduct optimal clinical decisions [26].

The systematic review and meta-analysis focus on vitamin D deficiency as a risk factor for DR. Vitamin D deficiency and risk of DR is a current hot topic because studies showed that patients with DR often have lower levels of vitamin D, which may increase the risk of developing and worsening the DR. This is explained with vitamin D’s anti-inflammatory and antioxidant properties that help protect the retina by regulating the growth of new blood vessels and reducing vascular damage, so a deficiency could contribute to microvascular complications in diabetes [27]. Indeed, patients with significant vitamin D deficiency (vitamin D levels below 20 ng/mL) have significantly increased the odds of developing proliferative DR by several times [28]. This systematic review analyzed data from 20 studies published up to October 2024 involving 22,408 participants and classified participants into groups with and without DR. Mean serum vitamin D levels were lower in patients with DR compared to those without DR, with a progressive decline observed across DR severity stages. In studies of patients with DR, vitamin D deficiency prevalence ranged from 27% to 95%. In everyday clinical practice, vitamin D deficiency must be considered as a modifiable risk factor in DR development and progression [29].

Finally, the Editorial of the first edition of the Special Issue “Molecular Research and Recent Advances in Diabetic Retinopathy,” comprising eight research articles and six review articles, presented the latest molecular research and recent advances in DR and DME [30].

In conclusion, this second edition of the Special Issue provides a comprehensive overview of recent advances and molecular research in DR in patients with type 1 and type 2 diabetes, aimed at improving our understanding and management of this vision-threatening complication. The new insights presented in this issue will contribute to earlier diagnosis and may serve as a basis for the development of future therapeutic strategies.
